# Intracranial pressure for clinicians: it is not just a number

**DOI:** 10.1186/s44158-023-00115-5

**Published:** 2023-09-05

**Authors:** Giada Cucciolini, Virginia Motroni, Marek Czosnyka

**Affiliations:** 1 Department of Surgical, Medical, Molecular Pathology and Critical Care Medicine, University of Pisa, Pisa, Italy; 2 Department of Clinical Neurosciences, Division of Neurosurgery, Brain Physics Laboratory, University of Cambridge, Cambridge, UK; 3 Institute of Electronic Systems, Warsaw University of Technology, Warsaw, Poland

**Keywords:** Intracranial pressure, Multimodality monitoring, Traumatic brain injury, Spectral analysis, Optimal CPP

## Abstract

**Background:**

Invasive intracranial pressure (ICP) monitoring is a standard practice in severe brain injury cases, where it allows to derive cerebral perfusion pressure (CPP); ICP-tracing can also provide additional information about intracranial dynamics, forecast episodes of intracranial hypertension and set targets for a tailored therapy to prevent secondary brain injury. Nevertheless, controversies about the advantages of an ICP clinical management are still debated.

**Findings:**

This article reviews recent research on ICP to improve the understanding of the topic and uncover the hidden information in this signal that may be useful in clinical practice. Parameters derived from time-domain as well as frequency domain analysis include compensatory reserve, autoregulation estimation, pulse waveform analysis, and behavior of ICP in time. The possibility to predict the outcome and apply a tailored therapy using a personalised perfusion pressure target is also described.

**Conclusions:**

ICP is a crucial signal to monitor in severely brain injured patients; a bedside computer can empower standard monitoring giving new metrics that may aid in clinical management, establish a personalized therapy, and help to predict the outcome. Continuous collaboration between engineers and clinicians and application of new technologies to healthcare, is vital to improve the accuracy of current metrics and progress towards better care with individualized dynamic targets.

## Background

Invasive intracranial pressure (ICP) monitoring is nowadays a standard of care in severe brain injury cases, as indicated by most recent guidelines [1]. Monitoring of ICP allows to derive cerebral perfusion pressure (CPP) and provides achievable targets for therapy, in order to avoid secondary brain injuries. Generally, in brain-injured adults ICP greater than 20–22 mmHg is defined as “high ICP” and demands active management [1].

Even if the single value of ICP remains important, a defined threshold of ICP is still object of debate, and the ICP signal carries many additional information about intracranial dynamics, retrievable either visually at the bedside or applying different computational techniques [2]. ICP trace can be used to (Table 1):

- Better comprehend pathophysiology of the injury and suggest targeted treatments (e.g., response to mean arterial pressure changes as suggested by the most recent Brain Trauma Foundation guidelines) [3, 4].
- Predict response to therapies [2].
- Forecast episodes of intracranial hypertension, using artificial intelligence algorithms, waveform morphology, or behavior of ICP in time [5–7].
- Predict the outcome throughout derived parameters (i.e., “ICP-dose” and “pressure-reactivity index”) [8–10].
- Provide information about cerebrovascular reactivity and optimal CPP (tailored therapy) [8, 11].

**Table 1:** Utility of ICP monitoring in clinical practice

Comprehension	Forecasts and predictions	Tailored therapy
Comprehend better pathophysiology of the injury and suggest different treatments.	Forecast episodes of intracranial hypertension. Predict response to certain therapies. Prediction of outcome.	Provide information about cerebrovascular reactivity and optimal cerebral perfusion pressure

ICP signal has been extensively explored during the last 50 years; techniques of signal analysis and artificial intelligence have been applied to ICP waveform and its trend. The published work includes animal and human experimental studies, mathematical modelling of intracranial components, as well as observational studies.

Many papers come from basic research and may pass unnoticed by clinicians. Thus, the aim of this narrative review is to summarize the recent research on invasive ICP monitoring to provide insights concerning (1) comprehension of intracranial pathophysiology, (2) outcome prediction, and (3) perspectives about tailored therapies and individualized thresholds of CPP and ICP.

## Comprehension of intracranial pathophysiology

### The Monro-Kelly doctrine and components of ICP: past and new insights

The balance between fundamental contents within the skull was first described in 1783 by Monro and is still considered valid. Assuming that the skull is a rigid and non-expandable box, Monro stated that the blood content within the skull should have been constant, so the amount of inflow should have equalized the outflow [12]. Kellie, gave a further contribution in understanding intracranial dynamic, including cerebrospinal fluid (CSF); he stated that any fluid contained in the cranium cannot be displaced without being replaced by another component, and that the same is valid if you introduce a new component into the skull [13]. When a displacement is not possible, any factor that provokes an increase in intra-cranial volume results in increased ICP.

$${\mathrm{V}}_{\mathrm{brain}}+{\mathrm{V}}_{\mathrm{blood}}+ {\mathrm{V}}_{\mathrm{CSF}}=\mathrm{K}$$

Where V_brain_ = brain volume, V_blood_ = blood volume, V_CSF_ = cerebrospinal fluid volume, K = constant

Each intracranial component can modify its volume in different ways and with a different time lag. Brain parenchyma is nearly incompressible; therefore, it is considered a static component, while blood and CSF are considered as “dynamic” as they can rapidly augment or reduce their volume [14].

### Arterial compartment

The arterial compartment can regulate cerebral blood volume (CBV) modifying vessels’ diameter (e.g., modulating cerebral blood flow, CBF). The amount of arterial blood in the skull can vary from 15 to 68 ml [15]. Regulation of CBF can act in seconds, with a mean time of reaction of 3–10’’ [16].

### Venous compartment

Blood outflow has been less studied but plays a crucial role in determination of ICP. Nearly 70% of the total amount of blood in the skull is venous, and ICP is directly related to central venous pressure (CVP) [14]. Behavior of the venous circulation is thought to be a passive reflection of arterial inflow because the eventually increased arterial inflow increases venous outflow. However, an imbalance between inflow and outflow may provoke a rise in ICP; in fact, pressure in the sagittal sinus regulates CSF reabsorption and is a major determinant of ICP [17]. Pressure in the venous system is influenced by downstream pressure, even if it is not passively transmitted: bridging veins within the skull act as a Starling resistor [18], preventing the retrograde transmission from CVP to ICP [14]. Research into the behavior of the venous compartment and the time lag of its compensatory mechanisms is highly awaited.

### Brain parenchyma

Brain parenchyma represents ~80% of the intracranial volume (1200–1600 ml); this compartment has a less static behavior than previously thought [19]. Some authors, proved that both neurons and glia may shrink, adjusting cell volume in response to different environmental stressors like pressure or osmotic changes. An experimental study by Kalisvaart et al. tested various models of intracranial damage in adult rats, to elucidate timing and extent of tissue modifications. After ischemic and hemorrhagic insults there was an increase in neuronal packing density and a reduction in cell volume diffused to many brain areas (even contralateral to the lesion), involving neurons and astrocytes [20]. The sum of apoptotic and pre-apoptotic shrinking in injured and non-injured neurons and glia, may alter the whole brain tissue volume and compliance, which changes dynamically over hours and days [21]; unfortunately, evaluation of the sole brain compliance remains nowadays a challenge.

### Cerebrospinal fluid

CSF has a volume of 1/10 of the brain (~150 ml), and regulation of its production and reabsorption is described by the Davson equation [22]:

$${ICP=P}_{CSF}={P}_{ss}+\left({R}_{CSF}\times {I}_{f}\right)$$

Where ICP = intracranial pressure, P_CSF_ = pressure of cerebrospinal fluid, P_SS_ = sagittal sinus pressure, R_CSF_ = resistance to CSF outflow, I_f_ = liquor formation.

In 1973, Marmarou expanded Davson’s work with a mathematical model explaining CSF formation, circulation and reabsorption [23, 24]. The model is based on the concept of capacitance and resistance, where capacitance is offered by the ventricles and resistance by the strictures in the CSF circulation.

The main determinant of CSF pressure is the sagittal sinus pressure, and CSF flow has a static and dynamic component (e.g., continuous and pulsatile flow, similarly to blood flow in arteries). One of the main roles of CSF is to distribute and equalize ICP; CSF compensatory reserve (e.g., the ability of CSF to absorb changes in volume without an increase in ICP) can be measured with infusion or withdrawal of fluid from the ventricles (see “Estimation of compensatory reserve with ICP” section for details) [25, 26]. Part of the CSF compliance has to be addressed to the lumbar sac, which has a relative capability of expansion in case of increased CSF pressure. In addition, other mechanisms of CSF reabsorption and displacement have been observed, such as filtration of CSF through nerve roots holes, and direct infiltration of CSF in the peri-ventricular brain tissue; this latter mechanism achieves CSF reabsorption by mixing CSF with extracellular fluid, that is directly reabsorbed into capillaries. This reabsorption mechanism has been called glymphatic circulation [27].

#### Conclusions and panoramic overview about raised ICP

Each compartment has its own dynamic behavior and co-participate to compensate an eventual rise in ICP. Every compartment has a different time of adaptation to changes, and different chemical and physical ways to do it. ICP represents the picture of elasticity and compliance of the whole system.

A panoramic overview of the main causes of raised ICP and their associated treatments is illustrated in Table 2 [28].

**Table 2:** Possible mechanisms of raised ICP that involve increase in one or more of the contents of the skull: blood (arterial or venous), CSF (hydrocephalus), or brain parenchyma (interstitial edema or tumor). ARDS: acute respiratory distress syndrome; SDH: subdural hemorrhage

**Compartment involved in raised ICP**	**Cause of raised ICP**	**Possible adequate treatment**
**CSF**	Increased production (rare) or reduced absorption	CSF drainage
**Blood**	Obstructed venous outflow (i.e. head positioning, sinuses thrombosis or compression, ARDS, prone positioning, abdominal compartment syndrome)	Repositioning of the head Adjust ventilation Neuromuscular blockage Venous thrombectomy or stent Anticoagulants Abdominal surgery
	Arterial vasodilation (impaired autoregulation)	Brief hyperventilation Vasoconstrictor drugs increase
	Bleeding	Appropriate treatment based on the source (i.e. coiling/clipping of aneurysms, evacuation and hemostasis for SDH)
**Parenchyma**	Interstitial edema	Mannitol, hypertonic saline, decompressive craniectomy
	Tumor/other mass lesions	Steroids Surgical excision

### Estimation of compensatory reserve with ICP

**Fig. 1:**
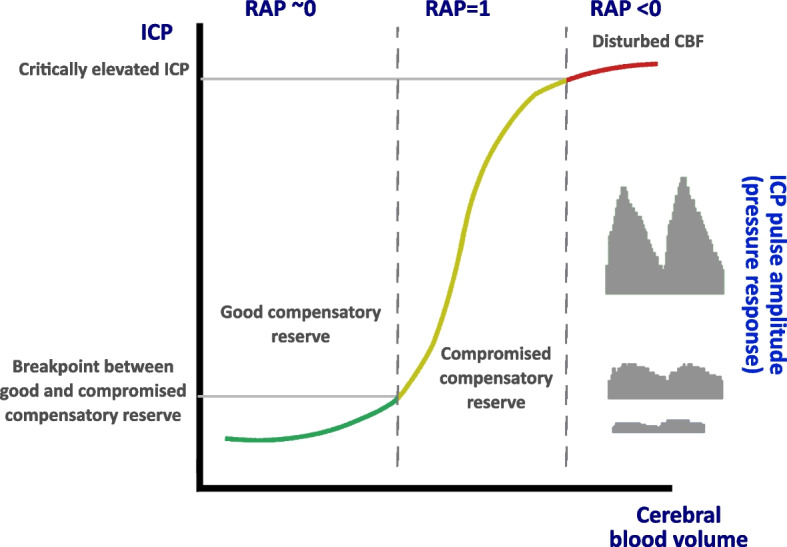
Hypothetical shape of cerebrospinal pressure-volume curve. For small increases in volumes (left part of the graph), pressure responds slowly and proportionally. This is a zone of good compensatory reserve: changes in volume produce low-pressure response. After the first breakpoint, ICP responds exponentially to a volume increase. This is an area of compromised compensatory reserve. Above a certain critical threshold of ICP (sources say that this threshold may vary between patients from 25 to 55 mmHg) the arterial bed starts to collapse and the curve tends to flatten, indicating exaustion of compensatory reserve along with decreasing CBF. RAP: correlation between amplitude and mean value of ICP (see text for details)

Cerebrospinal compensatory reserve is a general concept related to the contents of the skull and expresses the relationship between any increase in volume to an increase in pressure (Fig. 1) [29]. During the years researchers have been trying to plot the pressure-volume curve of the intracranial content, starting from the ICP signal. The relationship between pressure and volume defines the compliance of the system, and its inverse index, elastance. Compliance is the increase in volume provoked by an increase in pressure, while elastance is the change in pressure per unit change in volume (ΔP/ΔV) [30].

Quantifying elastance is clinically attractive as it should be predictive of impending exhaustion of the compensatory reserve. The volume-pressure curve of the brain describes a non-linear relationship with three distinct parts and slopes:

- At physiological volumes and low ICP, there is a linear rise in ICP with increasing intracranial volume. For small increases in volume, the ICP remains quite low, and the patient has a high compensatory reserve; small increases in volume can be compensated by a reduction in CBV or CSF displacement.
- Once the reserve is exhausted, a breakpoint is reached, and any subsequent increase in volume, increases the ICP exponentially.
- At high volumes, there is a change in pendency so the changes in volume are no more transmitted to changes in pressure, as this is already near the value above which a collapse in brain arterioles may occur. At this point, the patient has intracranial refractory hypertension and will go towards brain herniation if no intervention is performed.

### Estimation of dynamic compensatory reserve in research and at the bedside

Several approaches have been proposed to quantify the intracranial elastance at the bedside either intermittently or continuously. The first description of intracranial volume-pressure relationship was published by Marmarou et al. [6, 31]. He developed a mathematical model and introduced the pressure-volume index (PVI) defined as the notional volume (millimeters), which when added to cerebrospinal space, causes a 10-fold raise in ICP. PVI was calculated by measuring ICP changes in response to rapid injections or withdrawals of liquid from subarachnoid space. This metric has been used clinically [32]; however, due to the difficulty in standardizing the rate of volume change and the elevated risk of infection (needs to manipulate a ventricular catheter multiple times) this metric fell into disuse [33, 34].

Subsequently, a continuous index indicating the relationship between pulsatile CBV and ICP has been developed: the RAP index (R-symbol of correlation between A-amplitude of fundamental component of ICP and P-mean pressure) [35]. RAP is an index of compensatory reserve ranging from +1 to −1. When RAP is close to +1, there is synchronisation between the rise in mean ICP and its mean pulse amplitude (AMP), so a small rise in intracranial volume results in a high rise of ICP. A RAP value close to 0 indicates a lack of relationship between the changes in AMP and mean ICP. When RAP is −1 AMP has an inverse relationship with ICP (AMP decreases as the ICP continues to rise): at this stage, the compensatory reserve is exhausted and CBF falls (Fig. 1) [29, 36].

### Autoregulation estimation with pressure reactivity index

An estimation of autoregulation is possible throughout the ICP signal [37]. Autoregulation is an important autoprotective mechanism by which arterioles in cerebral vasculature dilate or constrict in order to maintain a constant CBF to the brain over a wide range of CPP (50–150 mmHg). After brain injuries autoregulation can be impaired, and continuous assessment of vessels reactivity might assist neurocritical care management [38, 39]. The pressure reactivity index (PRx) is a simple correlation coefficient between 10 s averaged ICP and ABP that measures cerebrovascular reactivity by observing the ICP response to spontaneous oscillations of ABP [8]. PRx measures the ability of arterial smooth muscles to respond to changes in transmural pressure [37]. A positive PRx means a positive correlation between ABP and ICP thus, passive behavior of CBV with a non-reactive vascular bed. A negative value of PRx reflects a normally reactive vascular bed (the ABP increase produces an inverse change in CBV and ICP). It has been demonstrated a tight and positive correlation between averaged PRx and the clinical outcome [40]; as PRx offers the possibility to calculate an optimal CPP, is possible to infer that targeting an optimal CPP in the context of a tailored treatment strategy might be possible in TBI patients. Nevertheless, PRx suffers from some weaknesses which should be kept in mind when interpreting this index. In some circumstances, it can be not reliable because it is based on the assumption that the only determinant of ICP variability is represented by an extracranial source, the ABP. On the contrary, brain’s arterial vasomotor tone can be influenced by other mechanisms of regulation of CBF, such as internal neurovascular adjustment and endothelial biochemical signalling. In addition, PRx may be unreliable in case of decompressive craniectomy (extremely high brain compliance), or during the application of external devices affecting simultaneously ABP and ICP (mimicking non-functioning autoregulation) [41]. Still, this index is extensively validated in many conditions, and it is the most widely used in clinical practice for continuous autoregulation estimation; improvement on its calculation, exploration of its pitfalls and a consensus on its use is awaited [42, 43].

### Pulse waveform analysis in time and frequency domain

The ICP pulse waveform can be analyzed in the time domain and the frequency domain. Each method is valuable and can give different information about ICP. Time domain is the most known by clinicians and available at the bedside, while frequency domain analysis requires techniques of spectral analysis (Fourier transform) and expertise, which may not be available in all centers. Nevertheless, frequency analysis is becoming popular for its capacity of adding useful information in real-time and a simple laptop at the bedside provided with proper software can easily perform such analysis (i.e., ICMplus^®^, Cambridge Enterprise Ltd., UK).

#### Time domain analysis

Time domain looks at the ICP waveform as it comes out from bedside monitors. Each ICP pulse waveform is generally composed of three peaks, strictly related to pulsation of ABP [2, 26, 44]:

- *P1 (percussion wave)*: caused by the distension of the walls of cerebral arteries transmitted from the aorta (synchronous and related to the systolic peak in ABP).
- *P2 (tidal wave):* related to the increase in cerebral blood volume. As cerebral arteries are compliant, the eventually increased CBV is mirrored by a delayed P2 in comparison to P1. P2 is more represented when brain compliance decreases.
- *P3 (dicrotic wave)*: it may represent aortic valve closure (synchronous with venous blood outflow) or a second peak of CBV.

Generally, P1 is related to cardiac ejection, while an increase in the arterial blood volume and its transportation is probably related to P2 and P3. Not all these peaks are always visible; nevertheless, P1 is normally dominant, followed by P2 and P3.

#### Normal and pathologic patterns

Peaks in ICP may change their proportions depending on cerebrospinal compliance, and there is a stepwise modification of the waveform with increasing ICP, as described by Kazimierska et al. [45].

**Fig. 2:**
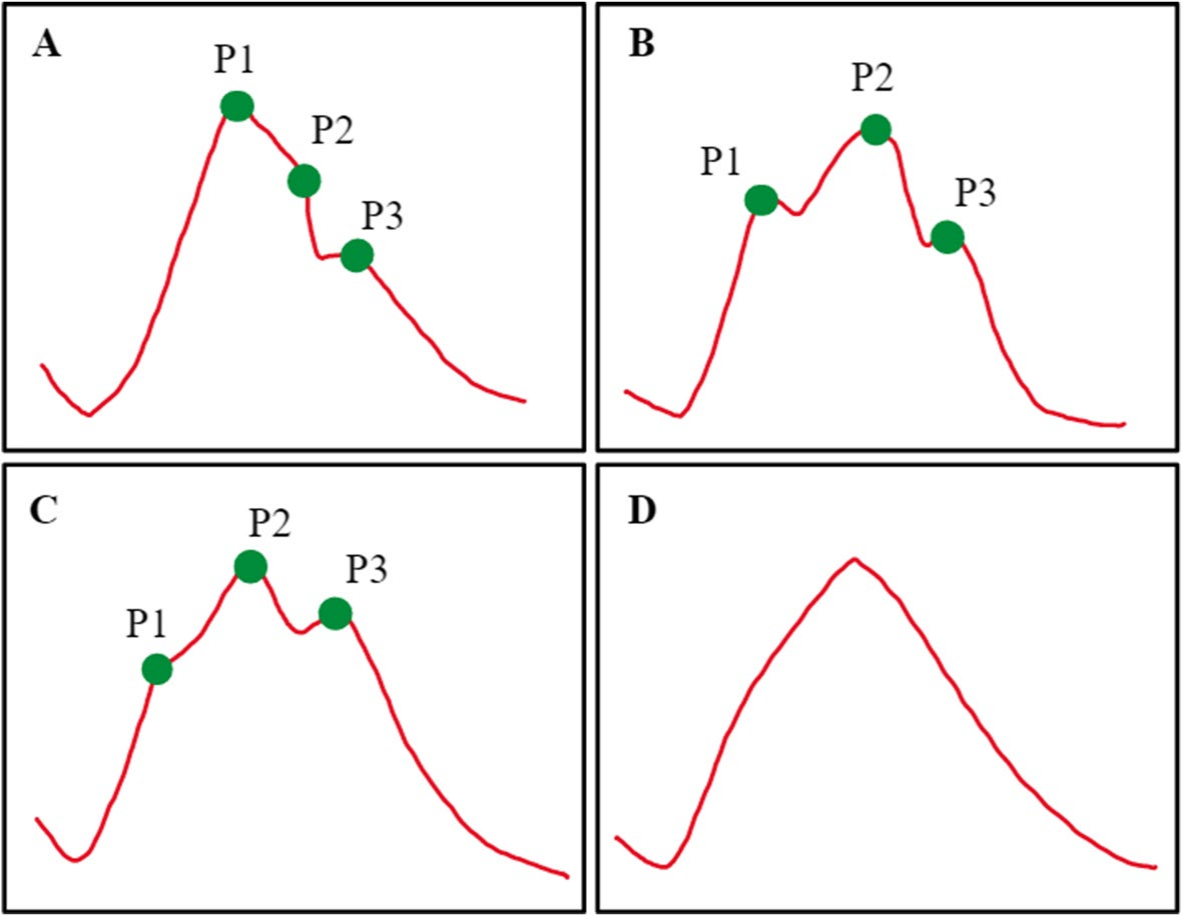
Pulse waveform analysis of ICP. Type A indicates normal ICP; there is a stepwise modification towards type D as intracranial compliance decreases. P1: percussion wave; P2: tidal wave; P3: dicrotic wave. See text for description

When brain compliance decreases, P2 increases and becomes predominant over P1 (type B waveform, Fig. 2). If the intracranial compliance is further reduced, then P1 becomes less visible and P3 approaches P2 (type C waveform, Fig. 2). The final stage is a “triangular-like” shape where the peaks are not anymore distinguishable (type D, Fig. 2).

Many metrics have been proposed to estimate brain compliance with waveform analysis. A well-known parameter is the P1/P2 ratio, that normally is > 1, and reaches values of 1 or < 1 in pathologic conditions [26].

Many research groups are working with algorithms that try to extrapolate an averaged pulse waveform from multiple series; once the mean shape of ICP is extracted, it is possible to calculate different metrics about cerebrospinal compliance. One example of this research projects is the MOCAIP (morphological clustering and analysis of intracranial pressure), a study in which 700 h of ICP recordings were analyzed with a proposed algorithm able to recognise non-artefactual signals and automatically distinguish the three ICP peaks, allowing further waveform analysis process [7, 46].

#### Frequency domain analysis

The process of decomposing a signal into its different frequencies is called spectral analysis and generates a power spectrum of that signal by applying the fast Fourier transform (FFT).

**Fig. 3:**
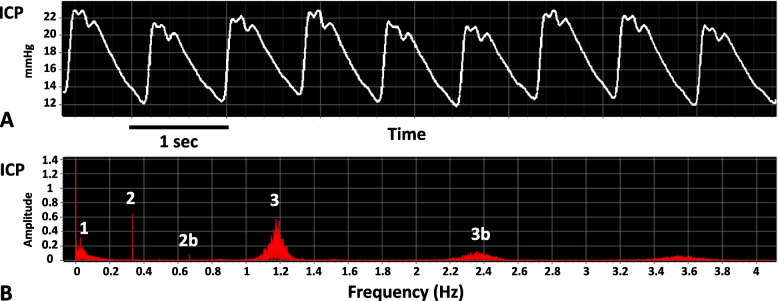
ICP in time domain (**A**), and frequency domain (**B**). Numbers represent: slow waves (1), respiratory waves (2), heart rate frequency (3). 2b is the second harmonic of the respiratory waves, and 3b the second harmonic of the heart rate

By decomposing the ICP signal, three main components can be identified: the heart rate (HR), the respiratory rate (RR), and other slow waves. HR is generally between 60 and 130 bpm, which translates into 1–2.16Hz, and is usually the most represented component, also called the fundamental harmonic of ICP. Respiratory waves are also well represented, and in patients sedated and ventilated the peak is usually very sharp and defined, as the respiratory rate is extremely regular (Fig. 3). RR is usually around 8–20 cycles/min, thus 0.13–0.33 Hz. In the slow frequency range, some waves that have a period of 20 s–3 min are represented; they are thought to represent cerebrovascular cyclic dilation and constriction in response to systemic haemodynamic variations or brain metabolism [47, 48].

### Behavior of ICP in time: during minutes (waves) and during hours (patterns)

During prolonged ICP monitoring, many types of waves have been observed. Based on the authors, different types of waves were described with different terms, and this contributed to generate confusion.

Overall, waves can be described by their frequency (period), amplitude (intensity), regularity, duration in time, and relationship with other waves. Among ICP waves we can recognize mainly: A-waves, B-waves, C-waves, and respiratory waves (Table 3) [2, 47, 49].

**Table 3:** Resume of the nomenclature and characteristics of the waves observable in ICP. A-B-C waves were first observed by Lundberg in the late 1950s and early 1960s. Subsequently, more studies involving continuous recording of ICP were published, and other nomenclatures appear

**Frequency range**	**Alphabetical Name**	**Alternative names**	**Descriptio**n
**Episodic, no frequency defined**	A-waves	Plateau waves	Characteristically shaped waves in ICP with three phases: ascending, plateau, descending.
**Slow waves, 0.005–0.05 Hz**	B-waves	Hyperaemic waves, vasogenic waves,	Very heterogeneous category, composed of slow waves with different morphologies: symmetric/asymmetric, with/without plateau waves superimposed, with/without ramps. Associated with increase in CBF and probably brain metabolism.
**0.1–0.15 Hz**	C-waves	Mayer waves or M-waves, Traube-Hering-Mayer waves	Sympathetic waves originating in the systemic circulation and transmitted to ICP.
**0.16–0.3 Hz**	R-waves	Respiratory waves	Waves synchronous with breathing.

#### Low frequency range waves

Slow waves of ICP have been extensively described. Slow waves represent ICP oscillations that have a duration of at least 20 s, up to several minutes (frequency of 0.005–0.05 Hz) [16]. These waves are generally repetitive but not regular, with variable amplitude. Because of their frequency, they occupy the left part of the ICP spectrum (Fig. 3), and they are thought to be the expression of vasodilation and constriction peculiar of the brain vessels.

##### B waves or hyperemic waves

**Fig. 4:**
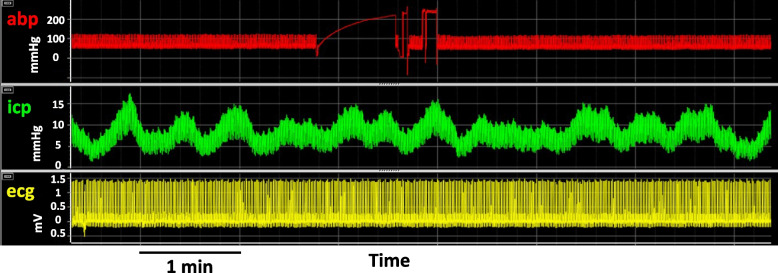
B waves in ICP. abp: arterial blood pressure, icp: intracranial pressure; ecg: electrocardiogram. It is possible to see that icp shows fluctuations (B-waves) while abp and ecg show not. In abp it is possible to se an artifact related to the flush of the arterial line

B waves were first described by Lundberg in the late ‘50 s [50]. They are long-lasting waves (from 20 s to 3 min), regular and repetitive, associated with changes in CBF, and they are probably associated with brain metabolism (Fig. 4). However, the term B-waves includes a lot of different subcategories based on symmetry/asymmetry, the presence of included plateau waves, and frequency; several authors refer to B-waves with different terminologies as slow B waves, or vasogenic waves [51].

During these kinds of waves the increase of CBF velocity and ICP is synchronised. The average magnitude of B waves after TBI is associated with the outcome: the grater the amplitude, the better is the outcome [29]. In addition, in awake patients with hydrocephalus, B waves alternate periods of silence and periods of regular waves. Usually, these waves appear when patients are in the REM phase of sleep, but they have been described in association with sleep breathing disorders outside of the REM phase [52].

### A waves or plateau waves

**Fig. 5:**
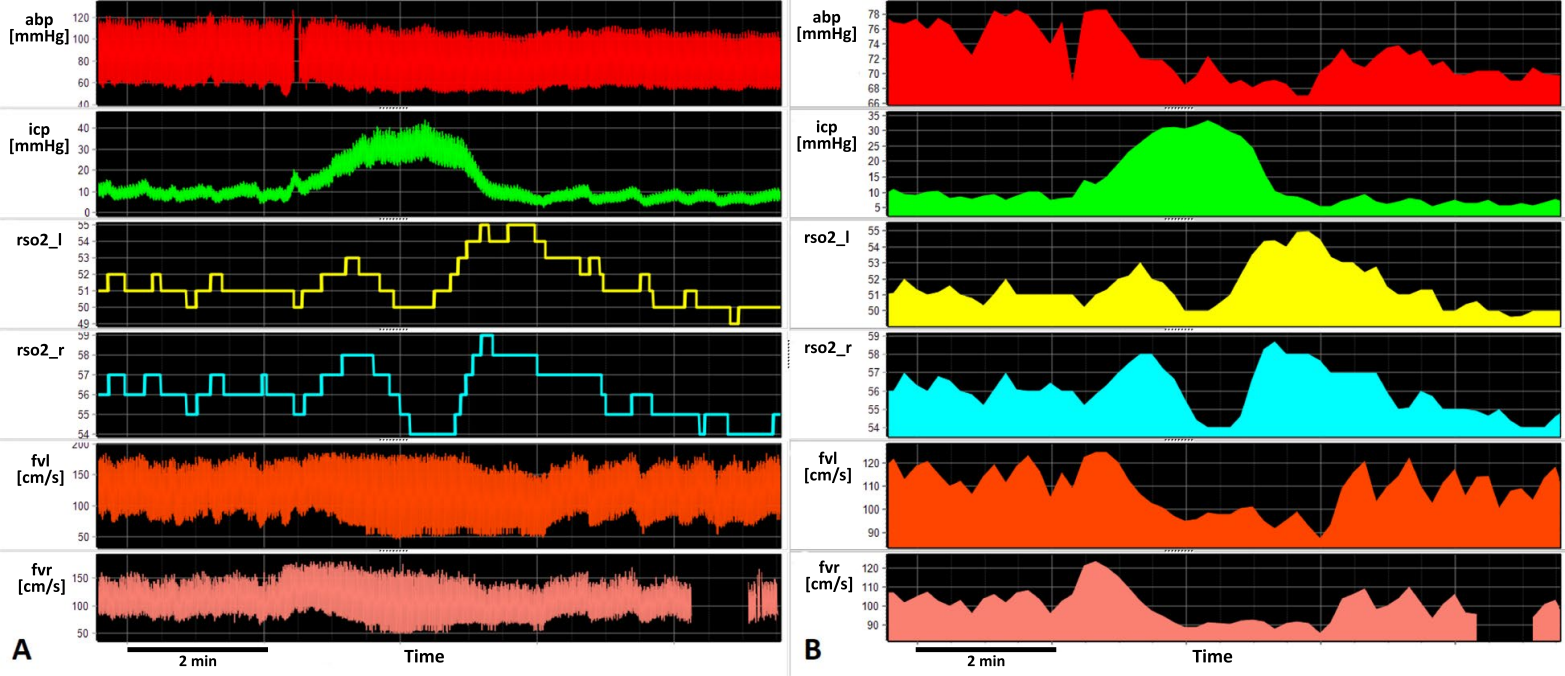
Plateau wave in a TBI patient. abp: arterial blood pressure; icp: intracranial pressure; rso2_l: brain oxygen saturation collected with near infrared spectroscopy, left side; rso2_r: brain oxygen saturation, right side; fvl: flow velocity left acquired with transcranial doppler; fvr: flow velocity right. On the left (**A**) raw signals collected during multimodal monitoring in ICU. On the right side, the same raw signals are represented as means over 10 s. While ICP increases, it is possible to see that flow velocity decreases in both sides, as well as the brain regional oxygen saturation

A waves, also called plateau waves, are represented by a sustained elevation of ICP that lasts for 5–30’ associated with a reduction in CPP and CBF. These waves are constituted by three phases: rise of ICP, plateau phase, and decrease of ICP (Fig. 5). These waves of ICP increase are caused by vessels’ dilation associated with impaired cerebrovascular pressure reactivity; cerebrospinal compensatory reserve is usually low. After the plateau phase, ICP usually drops below the baseline level and cerebrospinal compensatory reserve improves [29].

These kinds of waves are relatively common, and they occur in approximately 40% of TBI patients. Plateau waves are significantly more frequent in young patients, in patients with low midline-shift, low volume of contusion on CT scan, absence of skull fractures and low brain tissue concentration of carbon dioxide [53]. A waves may be observed as one-time transients or as a cyclic phenomenon; the mechanism of the plateau waves involves a “vicious cycle” which begins with cerebral vasodilation (i.e. as a consequence of a drop in ABP), resulting in an increase in CBV and ICP; the decrease in CPP provoked by increased ICP produces further cerebral vasodilation (vasodilatory cascade) which increases CBV and ICP [2, 54]. This occurs especially when autoregulation is working. Even though vascular resistance decreases, trying to augment CBF, this is not enough to outweigh the fall in CPP; thus, plateau waves represent a temporary brain hypoperfusion. Plateau waves usually terminate spontaneously after a few minutes, but this is not always the case [2]. It is recommended to terminate a plateau wave in 10–15 min by using any vasoconstrictor stimulus such as a brief period of hyperventilation or vasoconstrictor drugs [1]. Plateau waves lasting more than 30 min are in fact associated with worst outcomes in terms of mortality [29, 53].

### Respiratory waves

**Fig. 6:**
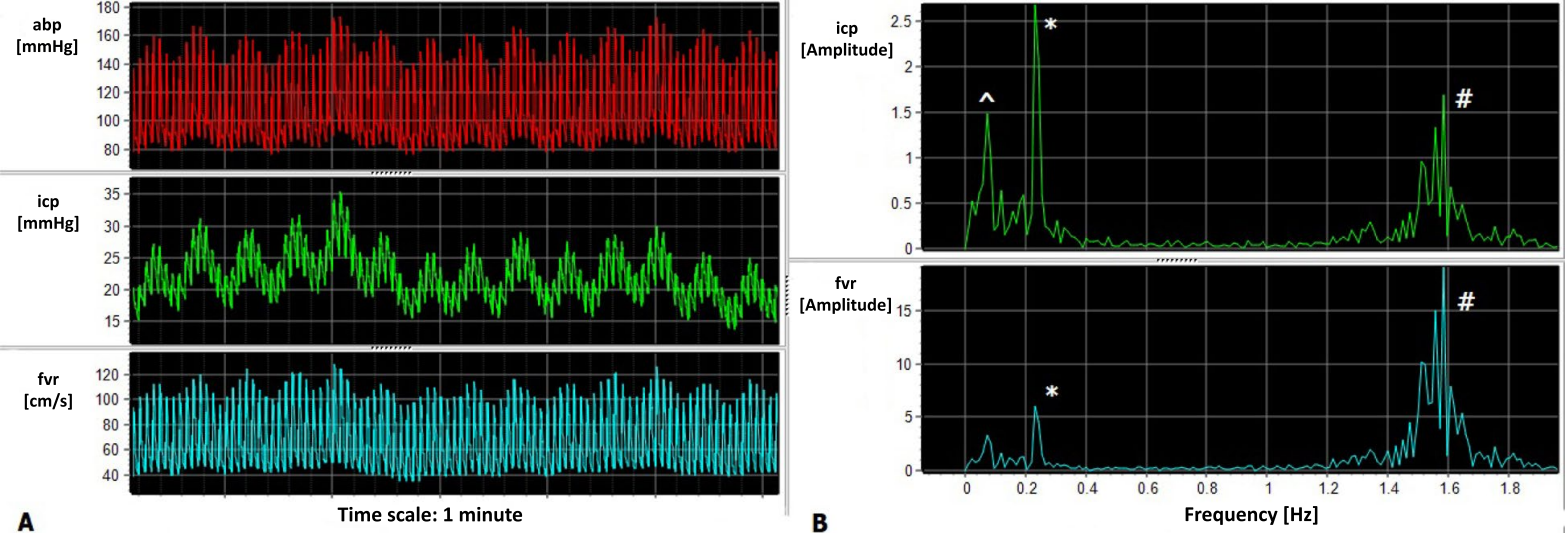
Respiratory waves present in arterial blood pressure (abp) and transmitted to intracranial pressure (icp) and blood flow in the right MCA (fvr). In **A**, the signals are shown in the time domain, while in **B**, there is icp and fvr represented in the frequency domain. Frequency of these respiratory waves is ~0.22 Hz, corresponding to about 13breaths/min. ^ represent the slow waves peak; * represents the respiratory waves peak. # represents the heartbeat peak

Respiratory waves are synchronous with breathing, so they have a frequency of 10–25 cycles/min (Fig. 6) [29]. Even if patients always breathe, these waves are not always visible in ICP. This kind of waves have been associated with increased resistance to CSF circulation in patients with hydrocephalus [55], and their amplitude is correlated with intracranial compliance in patients with hydrocephalus [56].

#### C waves o Mayer waves

Traube-Hearing-Mayer waves have a frequency of 0.1–0.15 Hz and are potentially associated with sympathetic nervous activity; they are thought to be linked to oscillations in baroceptors and chemoreceptors reflex control system. These waves originate in the systemic circulation and are transmitted with the modulation of cerebral autoregulation to intracranial vessels. Their amplitude has been proposed as a measure of sympathetic activity as it is determined by baroreflex gain and strength of the triggering [57].

#### Patterns of ICP behavior in patients with acute brain injury

Different waves and a baseline ICP can be combined in different ways in each patient, offering some distinguishable patterns:

- Low and stable ICP (lower than 20 mmHg);
- Low baseline ICP with plateau waves;
- High stable ICP (higher than 20 mmHg): could be the initial pattern after TBI;
- High unstable ICP (high ICP and plateau waves);
- Refractory intracranial hypertension: defined as a recurrent increase of ICP above 22 mmHg for a sustained period (10**–**15 min) despite conventional therapies [58]. The dramatic increase in ICP may cause brainstem ischemia and result in the Cushing reflex (or vasopressor response). In the first stage of the Cushing reflex, a sympathetic activation is triggered by the increase in ICP; ABP and heart rate (HR) rise trying to maintain an adequate CPP. In the second stage, the patient becomes bradycardic due to activation of baroceptors in the aortic arch consequent to the increased ABP. In the final stage, compression of the brainstem results in respiratory centers malfunctioning. This may be a preterminal pattern [59]. The pulse amplitude of ICP (AMP) starts to disappear before the terminal event [29].

## Outcome prediction

### ICP dose and CPP insults

The concept of ICP dose expresses the area under the curve for which ICP stays over a defined threshold and is expressed in mmHg/h. This method accounts for intensity and duration of intracranial hypertension and is thought to be an estimator of secondary brain injury. The ICP dose was demonstrated to correlate with mortality and functional outcome, and it is more sensitive when high-resolution data are used [9, 60].

ICP dose capacity of prediction has been demonstrated in TBI as well as in subarachnoid hemorrhage [61, 62]. The overall ICP burden can be evaluated at the bedside using continuous monitoring software such as ICM plus^®^ (Cambridge, Cambridge Enterprise Ltd., UK), and might give useful information about therapy strategies.

In addition, recent studies have investigated not only the ICP burden but also the CPP insults, demonstrating that CPP insults intensity and duration is related to outcome, and that the tolerance to CPP insults is different for different state of autoregulation and for the absolute mean ICP (threshold 25 mmHg). In a study by Guiza et al. [10], the low and high CPP insults were evaluated for intensity and duration and were plotted against the Glasgow Outcome Scale (GOS) using a color-coded scale. Interestingly, the tolerance to low and elevated CPP was higher if the duration of the insult was low. Episodes with ICP > 25 mmHg were associated with poor outcomes regardless of CPP. Patients with intact autoregulation better tolerated both higher and lower CPP insults.

The possibility to visualize ICP and CPP thresholds and duration and extension of the insult opens new perspectives about the identification of a personalized ICP and CPP threshold, and challenges the canonical concept of a universal and fixed ICP number that fits all [62].

### Prx and outcome

Many studies confirm the association between continuously measured PRx and global outcome in brain-injured patients [8, 39, 63]. Abnormal values of PRx indicate poor autoregulation and are associated with high ICP, low CPP, low GCS on admission and poor outcome at 6 months [37, 64]. Averaged PRx is an independent predictor of outcome after TBI; the critical value associated with increased mortality is approximately +0.25 [2, 40]. PRx well correlates with indices of autoregulation based on transcranial doppler and ultrasonography [2, 29]. When the lower limit of cerebral autoregulation is reached, PRx is strongly dependent on CPP and it increases with decreasing CPP. PRx has been used to calculate “optimal CPP” and guide therapies for patients with TBI; this could have a significant impact on mortality and outcomes in the next few years [64].

## Tailored therapy

### Optimal CPP and ICP

**Fig. 7:**
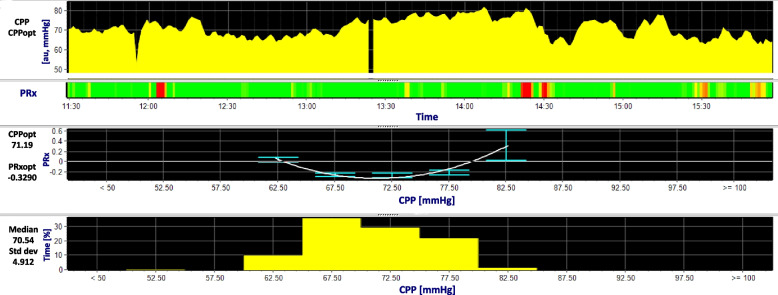
U-shaped curve for optimal cerebral perfusion pressure. CPP: cerebral perfusion pressure. PRx: pressure reactivity index. In the first time series is illustrated the mean CPP averaged every minute. PRx is shown as a risk bar chart, where green zones are indicating good autoregulation (PRx < 0), red zones are representing bad autoregulation (PRx > 0.3) and yellow areas are transition zones. When CPP is plotted against PRx, it is possible to evidence a CPP_opt_, that is the lowest point of the U-shaped curve, where PRx is the most negative. Crossing of the U-shaped curve with the x axis (PRx = 0) might represent the lower and upper limit of autoregulation. The graph at the bottom represents the distribution of the values of CPP for the period analyzed

Some authors demonstrated that in some patients the relationship between PRx and CPP might show a characteristic U-shaped curve [64]; this curve is obtained plotting CPP on the *x*-axis and PRx on the *y*-axis (Fig. 7). The U-shaped curve, if obtained, suggests that there is a value of CPP at which PRx is the lowest, so potentially a “best” CPP in which autoregulation status is the best for the patient. In this context, PRx can be used for the assessment of patient’s optimal CPP (CPP_opt_), which has been defined as the CPP at which PRx is most negative [65, 66].

Too low or too high CPP levels might be detrimental to the brain, potentially leading to ischemia or brain oedema. The greater the distance between the current and the CPP_opt_, the worse the outcome: when actual CPP is lower than CPP_opt_ there is an increase in mortality, when actual CPP is higher than CPP_opt_ there is an increased disability [2, 37, 64].

The concept of PRx-guided CPP therapy is also supported by the fact that brain tissue oxygenation increases with increasing CPP but only until the level of CPP_opt_; further increase in CPP does not improve oxygenation [67]. In addition, retrospective analysis showed that CPP_opt_ may vary individually, from 60 to 100 mmHg, so can differ dramatically from the guideline’s fixed thresholds (60-70mmHg) [4].

The CPPopt can be clinically estimated in real time by plotting and analyzing PRx-CPP curves in sequential 4-h time windows, to have a constant updated value for the CPP_opt_. Recent studies have updated the algorithm for CPP_opt_ calculation, using a multi-window weighted approach, to improve reliability and stability of CPP_opt_ calculation [68]. The CPP_opt_ Guided Therapy Assessment of Target Effectiveness (COGiTATE) study demonstrated the safety and feasibility of targeting the CPP_opt_ in TBI patients with ICP monitoring [11]. Nevertheless, PRx is under evaluation for its reliability and pitfalls, and new insights about its limitations and automatic recognition of unreliable raw data are awaited [43, 69].

## Controversies about ICP monitoring

ICP monitoring is considered a standard of care for brain-injured patients in many centres worldwide, even if an advantage of monitoring was never demonstrated. As indications for placing invasive monitoring of ICP are not clear, clinical practice between centres can be extremely heterogeneous [70]. Recent randomised controlled trials (RCT) have further ignited the debate; a multicentre RCT by Chestnut et al. conducted on 324 Bolivian and Ecuadorean patients investigated the efficacy of treatment based on monitoring of ICP vs standard treatment where ICP was not monitored [71, 72]. The authors showed no difference in the primary outcome (composite measurement of survival, impaired consciousness, functional and neuropsychological status at 6 months). Other observational studies comparing outcomes of patients in centres that use ICP monitoring have controversial results, with some authors reporting a better outcome, and others showing no differences [73]. In addition, as ICP monitoring implies intervention for targeting low ICP and an adequate CPP, a study by Cremer et al. showed increased levels of interventions, without an improvement in outcome [74].

Many confounding factors can influence results of these observational studies, as differences in treatment between centres and bias of selection, with exclusion of either the most or less severely injured. For this reason, while more RCT are awaited to clarify the role of ICP monitoring, the current brain trauma fundation guidelines still recommends to monitor ICP in all severe trauma with a level of evidence IIb, in order to reduce in hospital and 2 weeks post injury mortality [4].

## Conclusions

ICP monitoring guides the management of acute brain-injured patients in many centers worldwide, even though some controversies about its use are still ongoing. While a well-defined threshold for interventions has not been established, a lot of information is retrievable from this signal, which is characteristic for each patient and changes in time. The use of a bedside computer can implement standard monitoring giving clinicians new metrics to use at the bedside, aiding in clinical management for a better prediction of outcomes, establishing a personalized therapy and having a better pathophysiology comprehension. Constant communication between engineers and clinicians is crucial for improvement of the accuracy of the actual metrics, and progress towards patients’ individualized care.

## Data Availability

Not applicable.

## Abbreviations

ABP: Arterial blood pressure
AMP: Mean pulse amplitude of ICP
CVP: Central venous pressure
GOS: Glasgow Outcome Scale
HR: Heart rate
ICP: Intracranial pressure
CPP: Cerebral perfusion pressure
CPP_opt_: Optimal cerebral perfusion pressure
CSF: Cerebrospinal fluid
CBF: Cerebral blood flow
CBV: Cerebral blood volume
FFT: Fast Fourier transform
PRx: Pressure reactivity index
PVI: Pressure-volume index
RCT: Randomised controlled trials
RR: Respiratory rate
RAP: Index of compensatory reserve
